# Soft bilateral filtering volumetric shadows using cube shadow maps

**DOI:** 10.1371/journal.pone.0178415

**Published:** 2017-06-20

**Authors:** Hatam H. Ali, Mohd Shahrizal Sunar, Hoshang Kolivand

**Affiliations:** 1 UTM-IRDA Digital Media Centre and Game Innovation Centre of Excellence Institute of Human Centred Engineering, Universiti Teknologi Malaysia Skudai Johor Malaysia; 2 Department of Software Engineering, Faculty of Computing, Universiti Teknologi Malaysia, Skudai Johor Malaysia; 3 Department of Computer Science, Liverpool John Moores University, Liverpool, United Kingdom; Nanjing Normal University, CHINA

## Abstract

Volumetric shadows often increase the realism of rendered scenes in computer graphics. Typical volumetric shadows techniques do not provide a smooth transition effect in real-time with conservation on crispness of boundaries. This research presents a new technique for generating high quality volumetric shadows by sampling and interpolation. Contrary to conventional ray marching method, which requires extensive time, this proposed technique adopts downsampling in calculating ray marching. Furthermore, light scattering is computed in High Dynamic Range buffer to generate tone mapping. The bilateral interpolation is used along a view rays to smooth transition of volumetric shadows with respect to preserving-edges. In addition, this technique applied a cube shadow map to create multiple shadows. The contribution of this technique isreducing the number of sample points in evaluating light scattering and then introducing bilateral interpolation to improve volumetric shadows. This contribution is done by removing the inherent deficiencies significantly in shadow maps. This technique allows obtaining soft marvelous volumetric shadows, having a good performance and high quality, which show its potential for interactive applications.

## Introduction

Rendering realistic volumetric shadows at interactive rates remains an open research issue [1]. In spite of considerable efforts to simulate the volumetric shadows did not yield convincing results until now. The results of previous researches generally could not give realistic appearance of this phenomenon under discussion. The challenge lies in determining the area that is blocked from the light source which increases the complexity of rendering equation. Therefore, exclusion this area from rendering equation when evaluating a light scattering may be leads to improve the performance [2] [3]. Furthermore, determining whether there is any line can be seen directly from a light source for each point of the scene is yet another challenge. Therefore, many researches have focused on finding efficient methods for tracing rays that are emitted from a light source would hit sample points along camera rays until up to viewer.

Usually, interaction of light at the points of a surface is computed, while the effect of light scattering in the participating media is an important as well and should be computed for each point along view rays. The conventional ray marching is one of methods that commonly used in rendering light scattering by accumulating the values of the samples along the view rays. Therefore, in order to determine if a light can be scattered at a point, should be seen from light source first of all. The lookup in 2D height field is used for achieving this process where scattering occur along a ray from surface to the viewer. Whereas some radiance that travels along the ray is absorbed or out-scattered, the participating media can redirect in-scattered radiance, so that extra radiance reaches to viewer along same view ray. This redirected radiance comes from the overall light scattering by the participating media. Based on this assumption, the proposed techniques have still inefficient in real-time. Therefore, a careful mimic is required to meet the realism and provide phenomena close to real world. The volumetric shadows are one of these phenomena that appear as result to exist blocker in a scene which synchronize with interaction of the light within participating media. This is a deeply visible phenomenon and it often satisfies artistic requirements.

Many applications used models for rendering the effect of volumetric shadows which provide realistic images in many diverse fields, such as Earth browsers like Google Earth, flight simulators, Nasa WorldWind, Celestia, medical imaging, gaming and movies industries, entertainment, and safety-oriented research like the visibility of traffic signs in a foggy weather or exit signs in a smoke-filled room [4] [5] [6]. On other hand, in augmented reality applications, the volumetric shadows appear more realistic by combining RGB information and depth [7] [8], enabling the virtual effects to be seamlessly blended with real world. Furthermore, for mobile visual applications this effect could be increase quality of synthesized images [9] [10].

The brute force methods could compute all points, including invisible points of the light source in space of the samples along an entire view rays. That means, they compute all the samples including that are blocked from light source. Consequently, the exclusion for parts of the sample points from calculation on view ray, which block from a light source due to presence of obstacle in a scene is issue in extremely important. Shadow maps can be used to determine a set of points that blocked from light source within cube shadow maps. The main idea is to accelerate rendering by determining certain points to evaluate light scattering instead of all points of view rays such as what brute force methods do. This research aims to produce high quality of soft volumetric shadows by proposing SoftBiF-VS method, specifically at their boundaries with performance high enough for real-time rendering. SoftBiF-VS involves two important aspects. Firstly, it determines downsample points in ray marching method. Secondly, the scene is rendered from point of view of the camera by bilateral interpolation for obtaining soft volumetric shadows.

This technique has two main benefits. First, the bilateral filtering makes details of an image smooth, except the discontinuous depths. That means the effect of bilateral filter appears significantly at the edges of volumetric shadows. Second, this technique significantly reduces the number of sample points meanwhile benefiting from influence of bilateral filtering. This technique works efficiently and accurately to render soft volumetric shadows in real-time.

The immediate next section recapitulates related work. The third section, explores the methodology for achieving the research aim. The results and related discussions are presented in fourth section. The fifth section presents the conclusions and puts forward suggestions for future works.

## Related work

Introduced by Max[11], scan-line-based method to determine the brightness and darkness regions of atmosphere by contributing shadow volumes. Dobashi [12] proposed a solution to create volumetric shadows effect using sub-sampling. Although the results of volumetric shadows were acceptable for global illumination, this method created artifacts for local illumination due to sub-planes.

Biri et al.,[13]presented mathematical formulations for radiance transfer equation to simulate volumetric shadows based on shadow volumes and fog. However, these methods exploited capability and capacities of graphics hardware. Although efficient, the effects of volumetric shadows did not appear accurately. This was because the variation in participating media density did not coincide with variation in scattered light.

Wyman, C., & Ramsey, S. [14] their approach for rendering the effects of volumetric shadows induced scattering of light. This method combined sub-sampling and geometry-based shadows to accelerate the brute force algorithm. The ray view that contributed in scattering was computed using sub-sampling method. Then, geometry-based shadows were applied to determine lit and unlit space. However, the geometry-based shadows were impractical for complex scenes.

T´oth et al., [15] proposed a method to reduce the number of samples based on the relationship between a pixel and its nearby neighbors to compute the results of light scattering of each other.

Engelhardt et al., [16] proposed a method to compute intensities of the radiance transfer at depth discontinuities along epipolar lines of image-space.Meanwhile other intensities were interpolated between the depths of discontinuities to reduce the number of samples. This method was prohibitively expensive for complex scenes, which was a considerable shortcoming.

Baran et al., [17] presented incremental integration method using epipolar rectification to get good performance. The shortcoming of this method was the appearance of artifacts. Chen et al., [18] extended the incremental integration method by relying on 1D min-max mipmap. This method not only computed epipolar lines of the light, but also viewed rays to obtain a high performance. This method suffered from numerical stability issues when the light source existed inside the frustum of camera and needed to fall back to different approaches for such configurations.

Wyman C., [19] proposed voxelized shadow volumes method for visibility queries, which used epipolar space for downsampling of voxelize. This method was applying a parallel scan along view rays using bitwise OR, rather than adding operator to generate voxelized shadow volumes. The advantages of this method were: 1) separated geometric complexity, 2) reduce visibility costs, and 3) a few cache coherent lookups. However, this method inefficient with area light source appears an artifact and aliasing were the shortcomings of this method. Wyman C. & Dai, Z., [20]improved the above method in order to avoid the problems when using wide light sources.

Lin H. et al., [21] proposed a method to reduce the number of sample points for generating volumetric shadows in dynamic scenes. This method based on decision whether a pixel should be re-computed or used the information from previous frame. However, acceleration was reduced when moving objects and their geometry-based shadows contained a lot of screen pixels or the camera moved continuously.

Klehm O. et al., [22] proposed a method called rectify shadow maps to reduce samples instead of using brute force method. This method performed well and produced high quality outcomes. Nevertheless, this method was not successful when view and light rays were not parallel to each other, which meant the light source was directly invisible.

KlehmO. et al., [23] proposed a technique in order to add and edit holes into the shadow map of the generated shaft of light. This technique might serve artistic requirements based on the changing effects of light integral intuitively. Nevertheless, this technique did not provide realistic appearance of volumetric shadows due to incorrect information. This paper seeks to produce realistic boundaries of volumetric shadows as in real-world. Ali et al, [24] proposed SBFS method that based on bilateral filtering to generate soft shadow maps using multiple image-based algorithms.

SoftBiF-VS method is implemented for generating soft volumetric shadows. This techniquebased on the simulation of volumetric point light source. In real world, light sources always generate specific volumes[25] [26], However, the volume between occluders and shadow caster focusing in the shadow receiver is the critical part in shadows. Therefore, in this study we focused on the boundary of the shadow volume that develops realistic volumetric shadows. This effect is generated by compositing linear and volume of HDR, as well as interpolation using bilateral filter.

## Method

**Fig 1:**
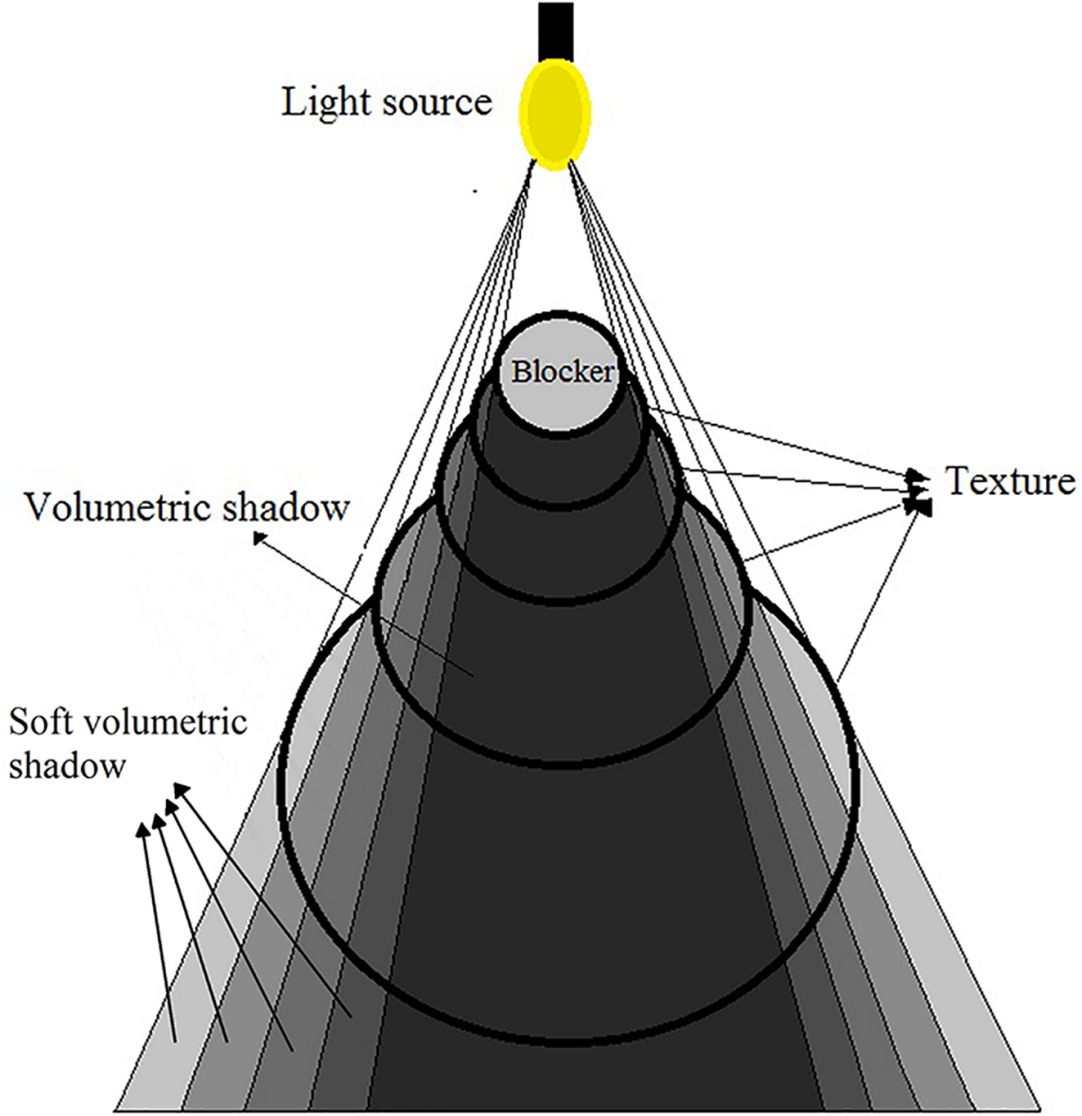
Volumetric shadow shows the umbra space, and soft volumetric shadow explains the appearance of the penumbra space.

Soft Bilateral Filtering Volumetric Shadows (SoftBiF-VS) is inspired from the appearance penumbra space at boundaries of the volumetric shadows when there is a blocker in front of the point light source Fig 1. The penumbra space is a border region of smooth transition between unlit and lit of the volumetric shadows. Usually, penumbra space at boundaries of volumetric shadows has smooth gradient with preserving-edges. Each edge represents shadow for the partially blocked point of the light points. SoftBiF-VS adopted this concept using cube shadow maps to cast soft volumetric shadows in all surrounding directions. Where each direction represents a view frustum of the light source for a face of the cube map. This view frustum will shifted slightly in number of the times to create sample points of the light. This points determined volumetric shadows at evaluating light scattering using ray marching method. Consequently, the volumetric shadows are processed using interpolate bilaterally of the pixels along and between edges.

SoftBiF-VS assumes set of light points in center of the cube shadow map, these points have view matrices which contribute in generating the shadows. The advantages of this technique is to produce evident soft volumetric shadows with developing overlaps between the edges. Furthermore, it allows removing the aliasing from boundaries that inherent in shadow map.

In short, this technique adopts the cube shadow map based on volumetric point light. Thus, the volumetric shadows are generated depending on the faces of the cube shadow map. The essential point of this technique is filtering to make the penumbra space correctly visible. This process is done by interpolated bilaterally to provide soft volumetric shadows. The following section describe this procedure.

### Light scattering model

In this research, the single light scattering model is used for generating volumetric shadows. SoftBiF-VS technique requires integrating each of lighting, attenuation, and scattering using ray marching along view rays. In this technique, downsampling is applied to reduce a number of sample points. The color of each sample point is evaluated for projecting on a pixels of the screen image. Consequently, the radiance that is redirected of the sample points on the view ray towards the camera to compute its contributions. In order to explain the scattering contribution, we start with optical depth as in equation.

$$D=e^{-tE*s}$$ (1)

Where *D* is an optical depth, *tE* is extinction coefficient of a texture, and *s* is a size of the step on the view ray. This formula is used to compute attenuation, which results in gradual loss in intensity of light scattering in participating media. Precisely, the attenuation process obtains scattered light on a view ray from an object to eye. The light scattering at each point compute as following equation.

F=l*s*tS (2)

**Fig 2:**
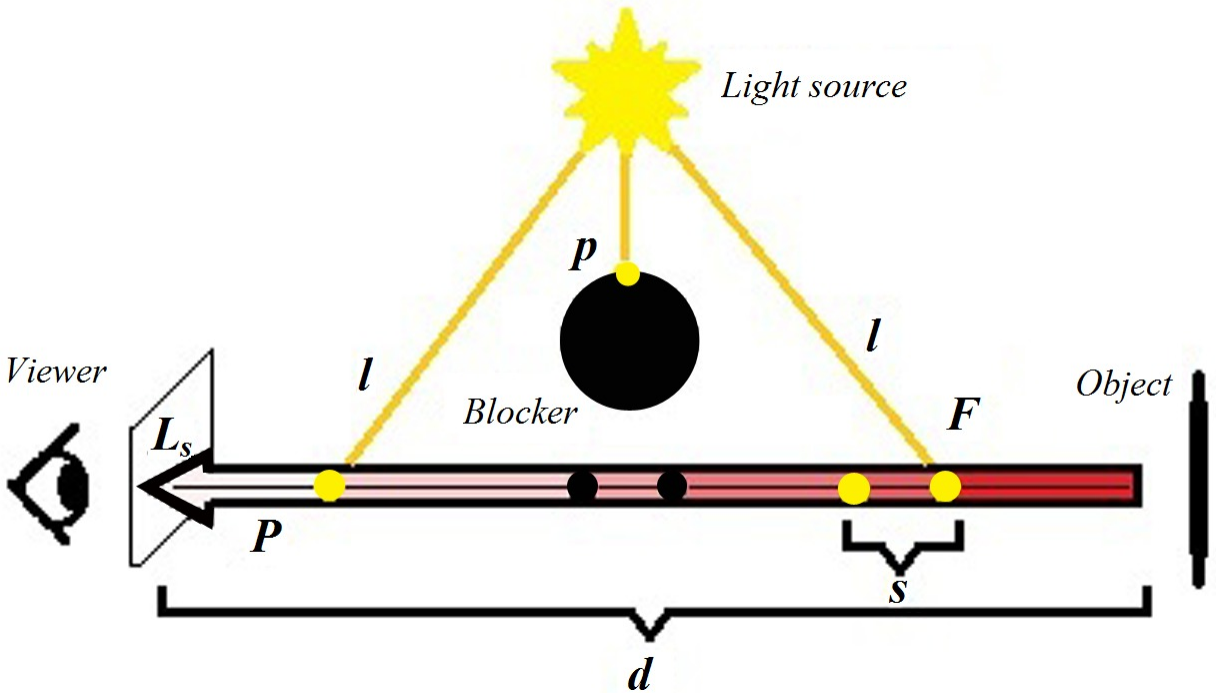
The parameters of light scattering model to compute radiation transfer equation.

Where *F* is a light scattered at a given point, *l* is a lighting computed lighting for each point along eye ray based on light position radiation and current position, and *tS* is the scattering of a texture. This equation is computed to determine light scattering for each sample point on the view ray. Fig 2 illustrates light scattering model with different parameters. The arrow indicates the extinction of light scattering shown gradation from red to white from object to viewer. The yellow points refer to the points that can be observed from light source. The black points indicate the shadow behind a blocker.

In fact, *U*_*sh*_, is computed from the depth map value for the current position.

$$U_{sh}=\left\{ \begin{array}{c} 1\;when\;P>p\\ 0\;when\;P\leq p \end{array}\right.$$ (3)

Where *P* is a light point depth, and *p* is the current position depth. *U*_*sh*_ represents visibility function that can determine whether a point can be seen from light source or not. Thus, the amount of light scattering *L*_*s*_ is computed that reach to eye according to the Eq (4).

$$L_{s}=\sum_{s}D*F*U_{sh}$$ (4)

The amount of scattered light for each pixel of the screen image is an accumulation of light scattering for all the sample points, multiplied in attenuation value along a view ray.

### Cube shadow maps

**Fig 3:**
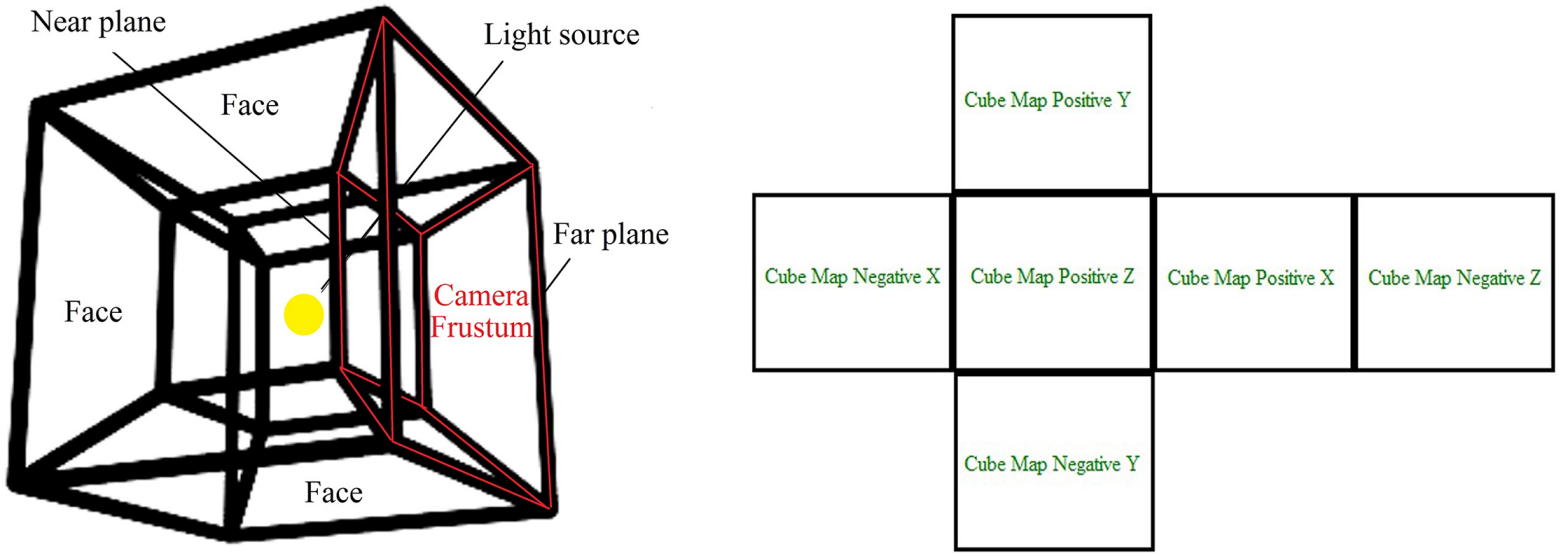
(Left)The near and far plane of a frustum within cube map; (Right)The six faces of cube map according to positive and negative axes.

In order to render the actual omnidirectional shadows, the light position is placed at the center of the cube map. Then, the near and far planes are determined for each frustum from camera position as illustrated in Fig 3 (left). The position of the camera can be updated depending on projection view matrix onto each face of the cube. These faces are represented in the axis X, Y, and Z in the positive and negative directions as illustrate in Fig 3 (right). The scene is rendered from the camera position onto off-screen using frame buffer object. The texture lighting is created using pixel shader in six directions for a cube shadow map faces using point volumetric shadow. The effect of shadows appears by sampling texture for each face of cube map, where graphics hardware plays an important role in constructing shadows.

### Bilateral filtering

Usually, when samples are reduced to compute light scattering that leads to appear artifacts as a result of so-called the downsampling. The problem of downsampling is concentrated on the boundaries between light and shadow, where is either in a region of light or in shadows, which means there is no smooth transition between their boundaries. In order to solve this issue, the bilateral filtering is exploited, which isone of non-linear filters that can be reconstruct smooth transition at these boundaries [27].

**Fig 4:**
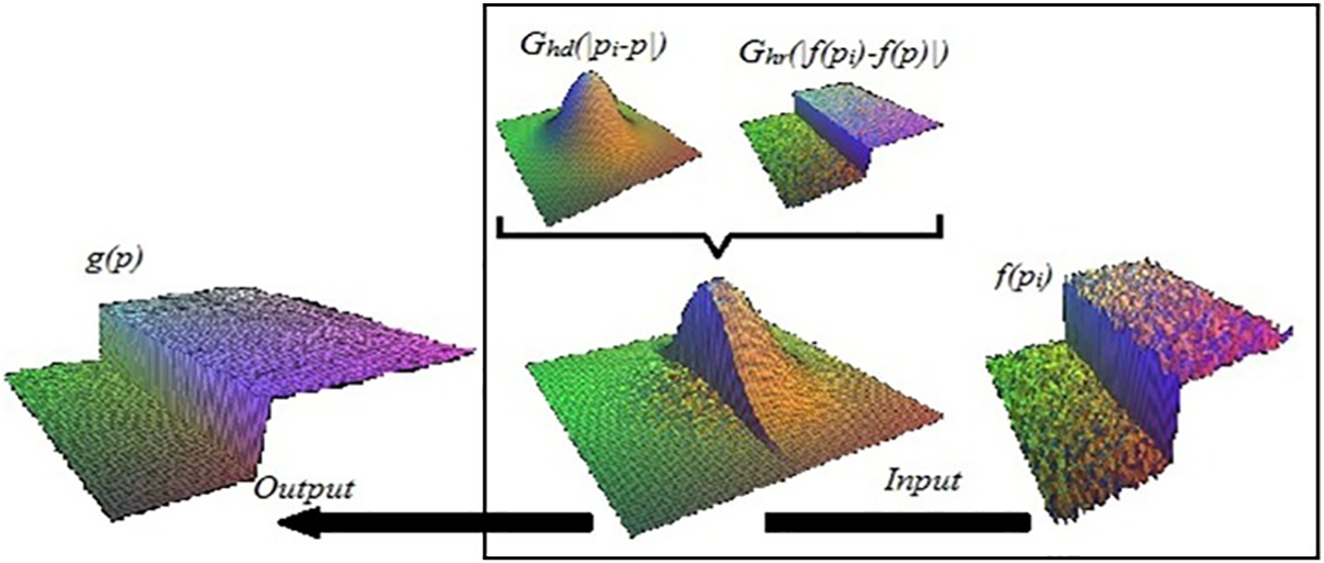
The parameters of bilateral filter on a height field.

The bilateral filtering successfully tackles the stochastic phenomena, where replaces the color value for each pixel of image by weighted average of color values for nearby pixelsas explain in Fig 4. It has a set of important characteristics, including edge-preserving, which is resulted from the differences between colors at shadows boundaries. In addition, it softens the regions between the edges within an image using Gaussian filter [28].

In summary, bilateral filter is a non-iterative edge-aware filter in which depends convolution by a Gaussian weight to contribute each pixel to result on a domain filter with ensure smoothing. Meanwhile, range filter that prohibits the influence of pixels of abruptly change in intensities, which computes as:

$$g(p)=\frac{1}{W}\sum_{p_{i}\in M}f(p_{i})G_{hr}(\left|f(p_{i})-f(p)\right|)G_{hd}(\left|p_{i}-p\right|)$$ (5)

Where *W* is normalization as:

$$W=\sum_{p_{i}\in M}G_{hr}(\left(\left|f(p_{i})-f(p)\right|\right))G_{hd}(\left|p_{i}-p\right|)$$ (6)

The range kernel *G*_*hr*_ is used for blurring differences in colors, where it contributes to reducing the effects of distant pixels to define the range filtering. While the domain kernel *G*_*hd*_ is used for blurring the differences in coordinates, it also reduces the influence of pixel *p* with a color value to represent the domain filtering. Parameters *h*_*r*_ and *h*_*d*_ are the measures for computing the filtering amount of image *g*for pixels’ locations and pixel values respectively. In this method, the image *g(p)* is filtered based on sampling for each pixel to create a smooth transition in regions of volumetric shadows. *f* is the original input image and its coordinates are centered in *p*. The current pixel *p* should be filtered and *M* is the mask. The weight *W* is assigned using the spatial closeness and the intensity differences[29]. In this method the weight is computed based on Gaussian distribution, as:

$$G_{h}(p)=e^{{-hp}^{2}}$$ (7)

*h* is a spatial extent of the kernel, size of the considered neighborhood, it must be adjusted to obtain comparable results. While *h*_*r*_ is the minimum amplitude of an edge, where the amount of the desired colors are set to achieve a combined of pixel values. That means both *h*_*r*_ and *h*_*d*_ can be controlled on results of the bilateral filtering. For instance, when *h*_*r*_ increases by the bilateral filter, it becomes more similar to Gaussian blur because the range Gaussian is flatter. While the domain parameter is increased, *h*_*d*_ becomes smoother.

The multiplied weights of the bilateral filtering have important features. This is because when none of the weights is close to zero, smoothing does not occur. In addition, the bilateral filter divides the input image into large-scale and small-scale components. The large-scale component considers anti-aliasing of the input to preserve the main edges. While the small-scale component considered residual of the filter, which can be using as a texture or noise by interpolated.

### Soft volumetric shadows

As the main target, SoftBiF-VS presents a new approach to improve the boundaries of volumetric shadows for pixels on the screen space. The interpolation bilaterally is applied in cube map shadow at shadows boundaries due to each pixel in its depth corresponds to pixel in the depth of the camera. Consequently, the bilateral filtering influence the image, especially along and between the edges of the samples of the penumbra space.

**Fig 5:**
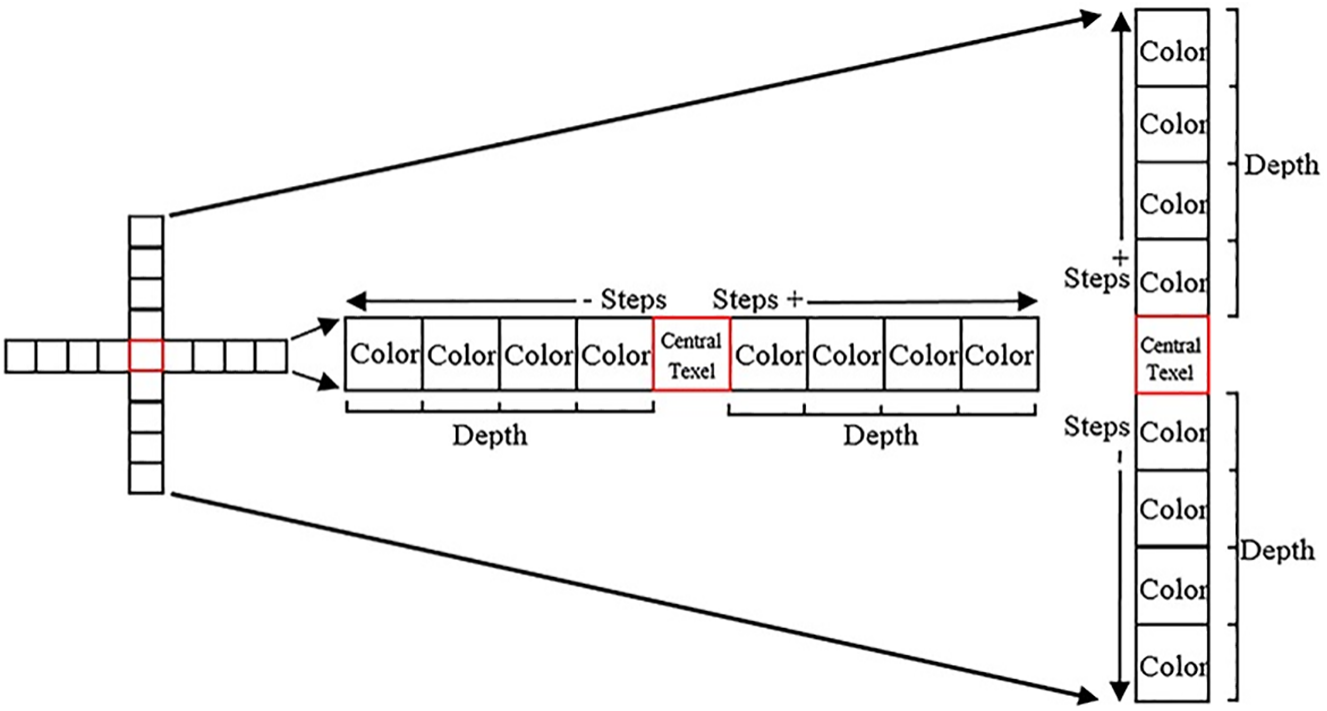
Bilateral filtering process in two 1D.

In this technique, the mechanism of action based on two 1D bilateral interpolation instead of 2D bilateral filtering to reduce of the cost. That means the filters used one after the other, filter each image row and then each column. When the color and depth of the central texel is determined, then moves by steps toward row and column to accumulate values in two directions the positive and negative respectively from a central texel, these steps are called taps. This process leads to smooth transitions in vertical and horizontal direction with preserved on lose crisp edges. Fig 5 illustrates the bilateral filtering process in vertical and horizontal direction. The steps of filtering process consist of one central tap and eight other taps, four on each side of a central pixel in both directions vertical and horizontal.

In the case above, the complexity becomes *O*(|*S*|σ_s_) instead of *O*(|*S*^2^|) since neighborhoods are replaced from 2D into 1D, where |*S*| is size of spatial domain. The results of this approach significantly faster than 2D bilateral filtering. As well as, this technique computes an axis aligned separable approximation of the bilateral filter kernel. Thus, this technique enhances the quality of the result, specifically on slanted boundaries, but arithmetically more included since the 1D bilateral filters are not axis-aligned.

**Fig 6:**
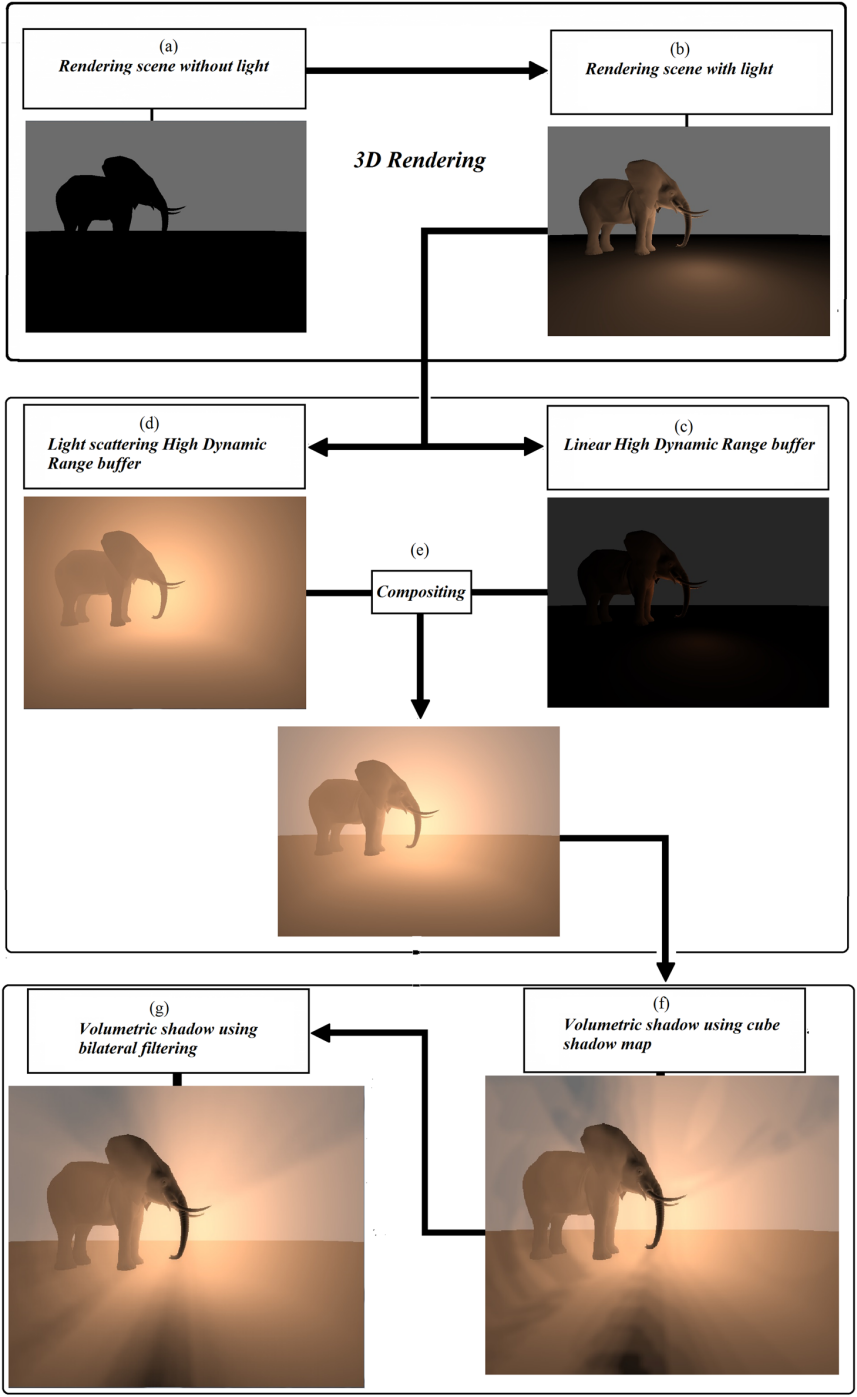
Visual framework of SoftBiF-VS, (a) Original input image without lighting, (b) with lighting, (c) HDR linear, (d) HDR volume, (e) compositing between (c) and (d), (f) rendering of cube map shadow, and (g) rendering of bilateral filtering.

Fig 6 illustrates the visual framework of the SoftBiF-VS method for generating soft volumetric shadows at the boundaries of volumetric shadow by rendering 3D scenes. The scene in Fig 6(A) represents the original input image without lighting. Fig 6(B) shows the scene with lighting using pixel shader in frame buffer object. Fig 6(C) rendered the scene using linear high dynamic range buffer. Fig 6(D) rendered the scene using volume high dynamic range buffer to generate light scattering. Fig 6(E) shows the compositing results of (c) and (d) using pixel shader in frame buffer object in order to obtain better images of light scattering. Fig 6(F) rendered the scene with cube shadow map that made the effect of volumetric shadows obvious. Fig 6(G) used bilateral filtering in order to obtain soft volumetric shadows. The downsampling and bilateral filtering could generate smoothed boundaries with crisp edges. The final image has an effective visualization of soft volumetric shadows.

The smooth transition at boundaries volumetric shadows lies beyond the appearance of the soft volumetric shadows. In other words, focusing on smoothing area between these boundaries meanwhile preserving edges could improve the appearance of the soft volumetric shadows. Nonetheless, this process is prohibitively expensive. In order to overcome this issue, interpolated bilaterally was employed to obtain better result by using downsampling. In this case, the soft volumetric shadows were simply generated, which have high quality and the process performs well based on a simple algorithm, which is explained in the following.

#### SoftBiF-VS algorithm

**Fig 7:**
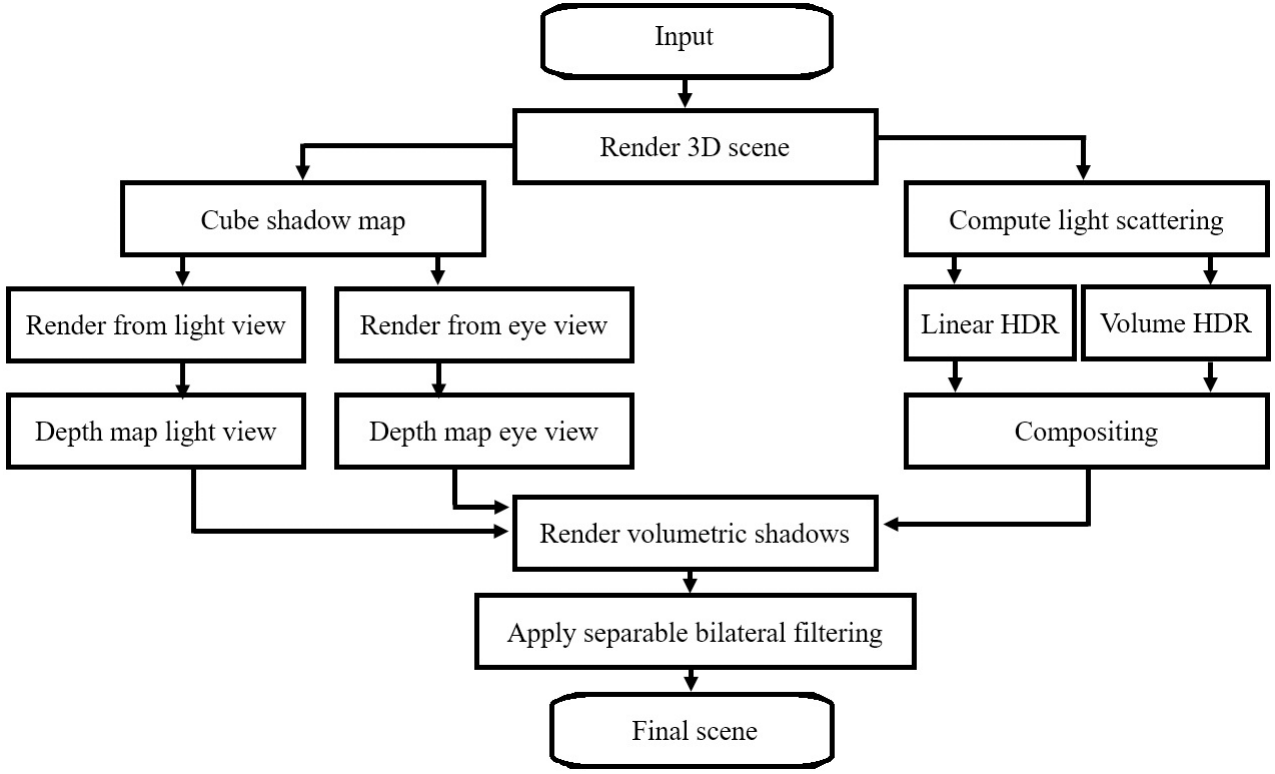
Process of Soft Bilateral Filtering Volumetric Shadows (SoftBiF-VS).

SoftBiF-VS is based on downsampling of the ray marching to generate light scattering for each pixel. It is composited from linear HDR and volume HDR to give more luminance to light scattering. The cube shadow maps play an important role in the appearance of volumetric shadows in different directions. Then, bilateral filtering contributes in eliminating artifacts effect. The result of this process is a smoother image with crisp edges. The flowchart in Fig 7 illustrated these steps.

The effect of this method can be observed as a result on the boundaries of volumetric shadows, which is soft volumetric shadows. Overall, in order to render a 3D scene with triangles volumetric point light and image screen are required. Moreover, the view camera should be taken into consideration. The proposed algorithm that renders soft volumetric shadows is presented in Algorithm 1.

**Algorithm 1. Soft Bilateral Filtering Volumetric Shadows (SoftBiF-VS)**

**Step 1.** *Rendering 3D scene with lighting using pixel shader*

**Step 2.** *Rendering scattered light*

- Compute linear HDR and
- Compute volume HDR and
- Compositing between linear HDR and volume HDR

**Step 3.** *Generate cube shadow map*

**Step 4.** *Rendering volumetric shadows*

**Step 5.** *Applying interpolation using bilateral filter*

**Step 6.** *Rendering the scene with soft volumetric shadows*

At first, the main scene was rendered using a frame buffer object, which involves color and position of the light. The lighting is created using pixel shader based on sphere light to produce lighting without light scattering.

The light is rendered of scene and evaluating light scattering with taken into account the presence of an occluder and participating media. Both types of high dynamic range HDR are used, the linear HDR and volume HDR to improve a lighting of the scene. Where linear HDR is computed depending on multi texture coordinates the derives from high resolution of scene, while the volume HDR is computed using bilinear weights. This step is considered as the first layer to calculate scattered light scattering with high quality.

Cube shadow map is created by using framebuffer object and makinga light position at its center. In this case, it could determine the near and far plane for each face. The cube shadow map is updatable for six faces ofthe scene for each frustum. Moreover, the cube shadow parameters were computed, they contribute to compute shadows and lighting within cube.

**Fig 8:**
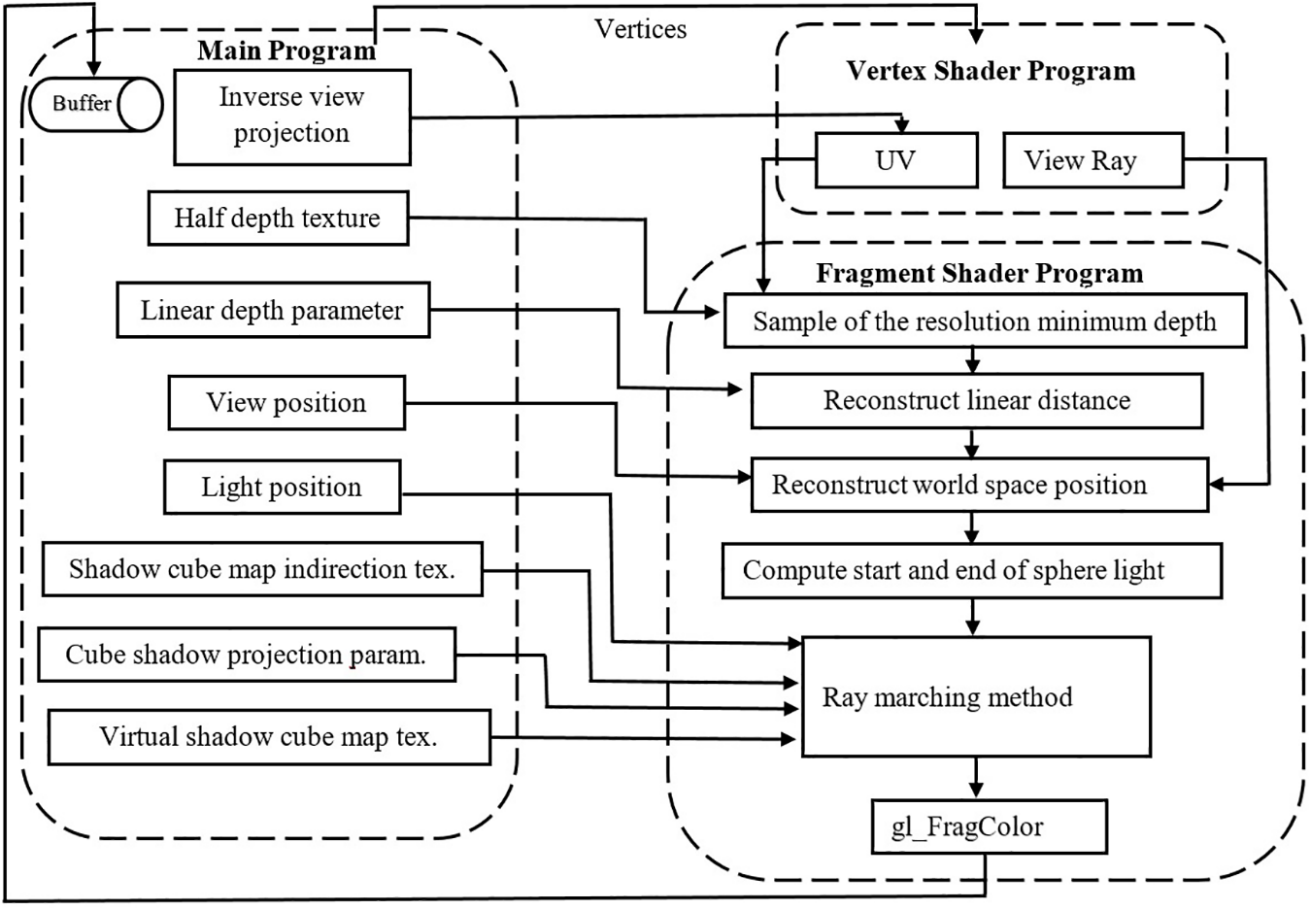
Procedure of vertex shader and fragment shader for input of main program (SoftBiF-VS).

A framebuffer object is used for rendering volumetric shadows in quad-screen. Basically, a comparison is performed between sample the virtual depth map value and the depth map value for the current position to compute a shadow. The latter operation contributes in providing volumetric shadow in the scene using vertex shader and fragment shader. The flowchart in the Fig 8 shows this procedure, also a more detailed processing is shown in Appendix A with define all the used variables.

Interpolation is applied to tackle the improvement issues like eliminating artifact. In this process, the bilateral filter proposed by [29] is used. The scene is rendered using two framebuffer objects as an off-screen. The first one isa temporal half resolution color buffer based on blur direction step to ward its width. The second one was a current volumetric buffer based on the blur direction step toward its height. The look up weight of bilateral blend is based on the center depth and sample depth. The taps represent the width and height in horizontal and vertical directions. This process leads to a smoothed image with preserved-edges.

Finally, SoftBiF-VS rendered the triangles of the objects in the scene with soft volumetric shadows using cube shadow maps. Textures are the important elements in computing the light scattering in participating media within the framebuffer object. Pixel shader is used to initialize volumetric shadows for filtering process. The generated soft volumetric shadows eliminated the inherent shortcomings in the shadow maps to optimize performance in real time and produce realistic image.

## Results and discussion

SoftBiF-VS is measured and implemented on a 2.5 GHz Intel(R)HD Core(TM) i5-3210 CPU using an ATI Radeon HD 7670M Graphics 4000 with 6GB of RAM. SoftBiF-VS is written in OpenGL and the shader was compiled with Shader Model (3.0). All scenes are tested at 800×600 resolution for rendering models.

As in many other fields of computer graphics, the acceleration of the soft volumetric shadows involves a tradeoff between rendering performance and quality enhancement. In other words, reducing sample points make the performance of soft volumetric shadows more efficient; nevertheless this would decrease the quality of appearance. Conversely, increasing sample points would reduce the efficiency of rendering and improves realism. In order to enhance the efficiency, the sample points is reduced for light texture with tackled using bilateral filtering.

**Fig 9:**
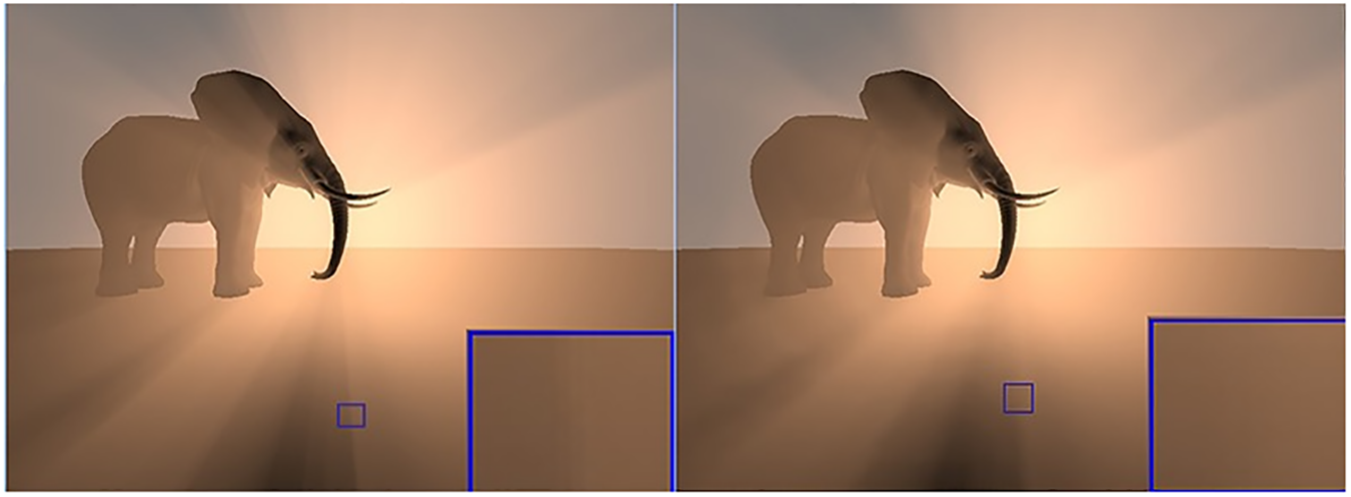
The two different scenes of the Elephant model, (left) rendering the scene using 200 step for each sample without bilateral filtering by ray marching, (right) rendering the scene using 60 steps for each sample with bilateral filtering.

Fig 9 shows two different situations for rendering a model Elephant, which needs six passes to be rendered in cube shadow map. The first situation, 200 steps are used for the sample to generate volumetric shadows without bilateral filtering. This represents traditional ray marching method as is illustrated in Fig 9 (left). It is clear that the volumetric shadows with sharp edges and the speed with15 FPS. The second situation used60 steps of the sample for generating soft volumetric shadows with bilateral filtering as can be seen in Fig 9(right). Soft volumetric shadows with crisp edges are generated with61 FPS. A comparison between Fig 9(left) and Fig 9(right) shows smoother at boundaries in (left).It also kept the edges homogeneous and crisp within the space of volumetric shadows. Arguably, Fig 9(right) is prohibitively expensive due to the use of more steps for each sample. Fig 9(left) does not require60 steps for each sample which increases the speed performance. This means, when bilateral filter is employed, using minimum number of sample points, the costs of rendering decrease, and at the same a pleasing and attractive result is produced.

**Fig 10:**
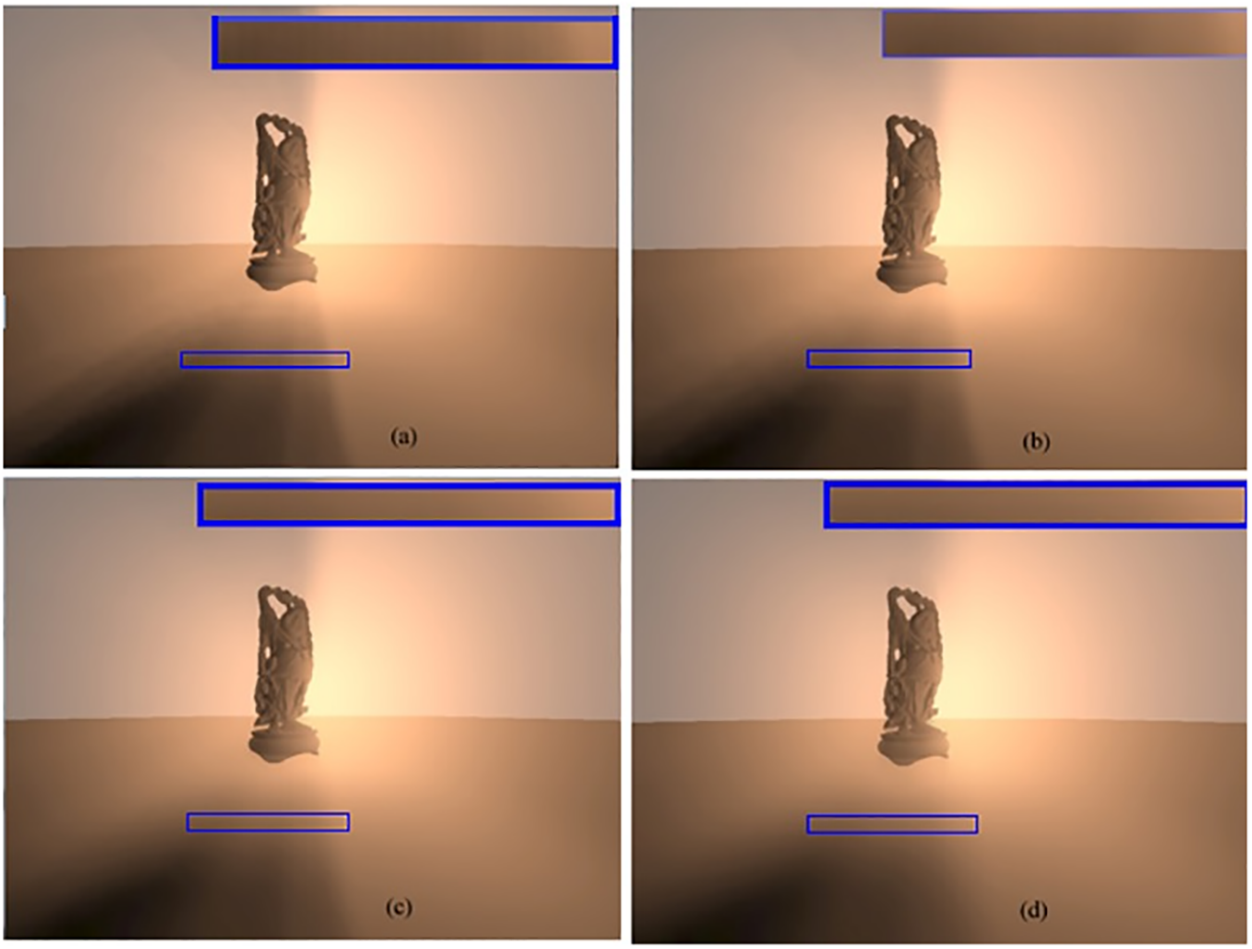
Buddha model using bilateral filtering, (a) the scene with 4 taps, (b) the scene with 8 taps, (c) the scene with 16 taps, and (d) the scene with 32 taps.

Fig 10 shows rendering of Buddha model in a scene containing volumetric shadows. It indicates the influence of the taps number on a texture of the ray marching using bilateral filtering. Since undersampling of ray marching develops severe artifacts, a minimum of steps are adjusted to suit the rendering time. Noticeable differences can be observed because of the effects of taps number t in Fig 10(A), 10(B), 10(C) and 10(D), which use 4 taps, 8 taps, 16 taps, and 32 taps, respectively. In these cases, the appearance of the scene got smoother whenever the number of taps increased. This was due to the increase in the current optical depth, which carried a small computational cost as the number of the samples increased. Nevertheless, the crisp edges remained within the soft volumetric shadows, which were realistic and eye-pleasing to viewers.

**Fig 11:**
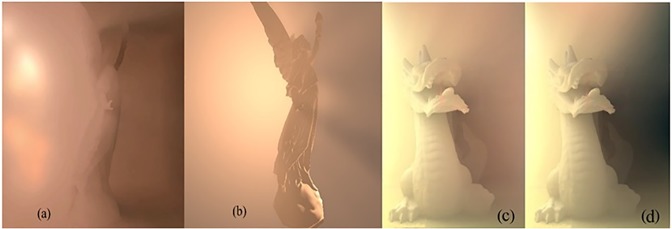
Rendering two different models using interpolation: (a) and (c) the imperfect results of Wyman and Dai’s VSV method [20], (b) and (d) the results of SoftBiF-VS method.

Fig 11(A) and 11(B) illustrate the results of rendering Lucy model (525000 triangles), while Fig 11(C) and 11(D) is Dragon model (871414 triangles). (a) and (c) are the results of Imperfect VSV method developed by Wyman and Dai [20], while (b) and (d) are the results of SoftBiF-VS method. The comparison between the two methods with interpolation appears in soft volumetric shadows reveals that SoftBiF-VS method performed remarkably in terms of quality from Imperfect VSV method [20]. On another hand, the performance of SoftBiF-VS for rendering Lucy and Dragon with54 fps and 46 fps, respectively. While the performance of Imperfect VSV with23 fps and 19 fps, respectively. It is obvious that the soft volumetric shadows in Fig 11(B) and 11(D) are clearer than volumetric shadows in Fig 11(A) and 11(C) specifically at Lucy's hands and the right side of the dragon.

**Fig 12:**
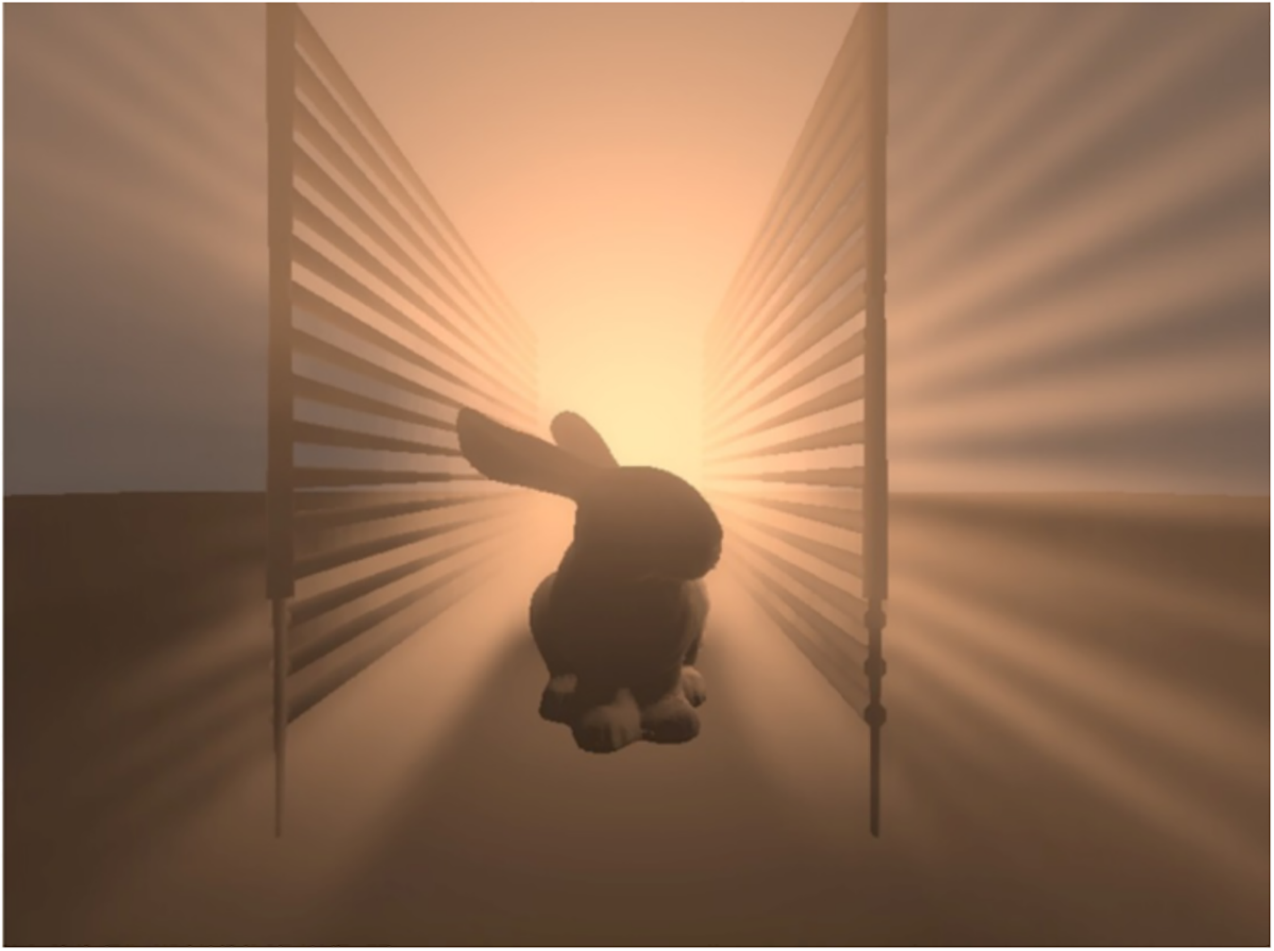
Bunny and Fence models on shadows: Right side: the effect of Fence model; Left side: the effect of Bunny and Fence together, bottom the effect of bunny on shading the scene.

Fig 12 demonstrates the ability of SoftBiF-VS method to present effective soft volumetric shadows. The scene made of Bunny and Fence model. It used volumetric point light in participating media. Clearly, the volumetric shadows spread in all directions along the light rays. At the right side of the scene, the fence influenced the scattering of the light, while at left side both the bunny and the fence affected the light. On the bottom of the scene, the influence of bunny on shading is also obvious. SoftBiF-VS method could produce the phenomenon of volumetric shadows with high quality and correct appearance.

**Fig 13:**
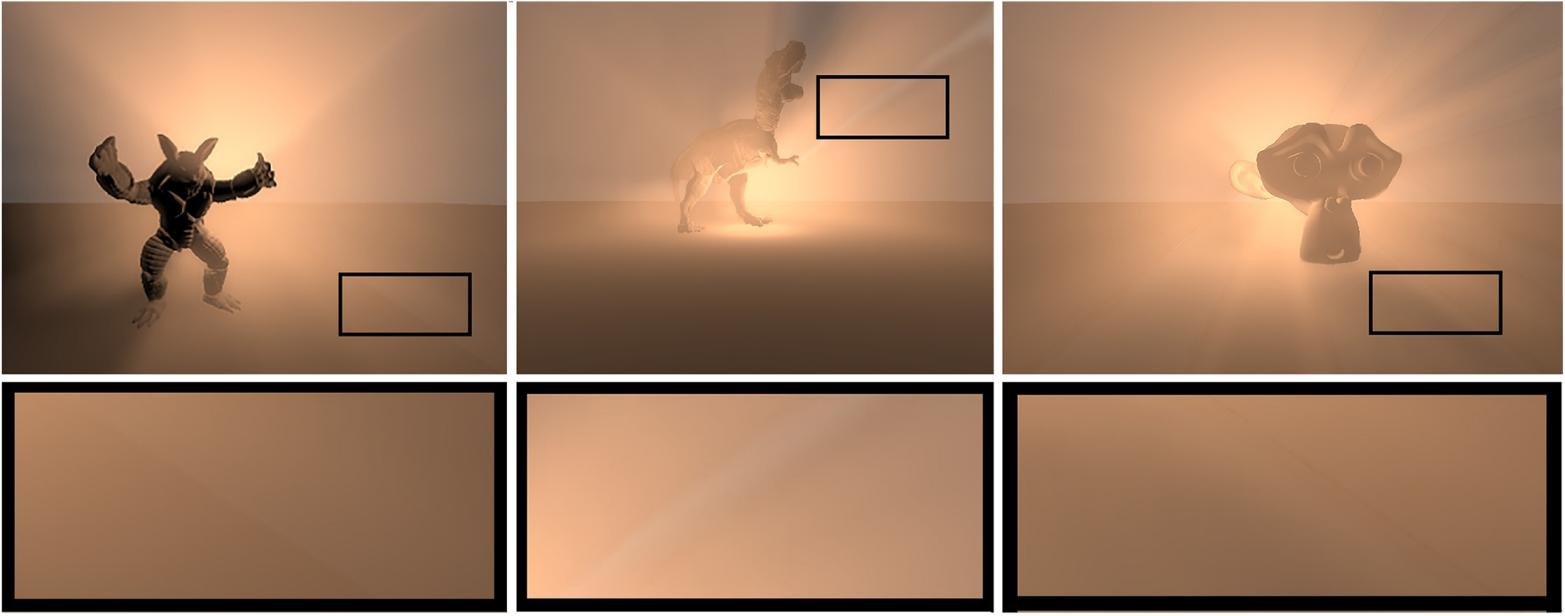
Shows effect soft volumetric shadows of our method using models in different sizes.

SoftBiF-VS method presents the proper appearance of the soft volumetric shadows, where light scattering in participating media spreads smoothly from volumetric point light. In fact, the appearance of soft volumetric shadows is based on the subtle effect that occurs at the boundaries of the volumetric shadows as illustrated in the bottom (black rectangles) Fig 13 for models of Stanford Armadillo (left) (212574 triangles), Stanford Tyrannosaurus (middle) (200000 triangles), and Blender's Suzanne (right) (15488 triangles).

Comparing the discussed models, it is apparent that the number of triangles has a small effect on the rendering frame-rate of SoftBiF-VS method. Particularly, while the number of triangles in a scene in SoftBiF-VS method is imperceptible, the performance remains within real time.

The important measures that control over the filtering amount of the SoftBiF-VS are domain parameter (*h*_*d*_) and range parameter (*h*_*r*_). For example, the increasing amount of *h*_*d*_ and hr lead to a blurry image, thus it gives good results that confirm 1) the improved soft volumetric shadows and 2) decrease the rate of frames. However, the upsample of light texture develops a more realistic soft volumetric shadows, but through a time-consuming computation process. The time increases because of the increasing number of required passes for rendering cube shadow maps. This discussion suggests that an accurate balance should be achieved between sampling, *h*_*r*_, *h*_*d*_ for rendering effective and efficient scenes.

Table 1 compares the run-time performance of Biri’s method [13], Wyman’s Imperfect VSV method [20], and SoftBiF-VS using downsampling of light texture and bilateral filtering. The models used in this research are Suzanne, Elephant, Bunny, Tyrannosaurus, Armadillo, Dragon, Lucy, and Buddha in 800*600 resolution. The SoftBiF-VS showed a better run-time performance compare to Biri’s method [13], Wyman’s Imperfect VSV method [20].Moreover, the high quality of soft shadows is remarkable.

**Table 1:** Run-time performance comparison betweenSoftBiF-VS,Biri’s method [13], and Wyman’s Imperfect VSV method [20] with different models.

Model	No. of Tri.	Biri, [3] (ms)	Imp. VSV (ms) [21]	SoftBiF-VS(ms)
Suzanne	15488	25.6	14.4	9.1
Elephant	39290	47.6	21.7	14.5
Bunny	69451	71.4	25.6	15.9
Tyrannosaurus	200000	83.3	32.3	16.9
Armadillo	212574	90.9	34.5	17.2
Lucy	525000	109.4	43.5	18.5
Dragon	871414	252.6	52.6	21.7
Buddha	1087716	410.5	79.9	27.03

**Fig 14:**
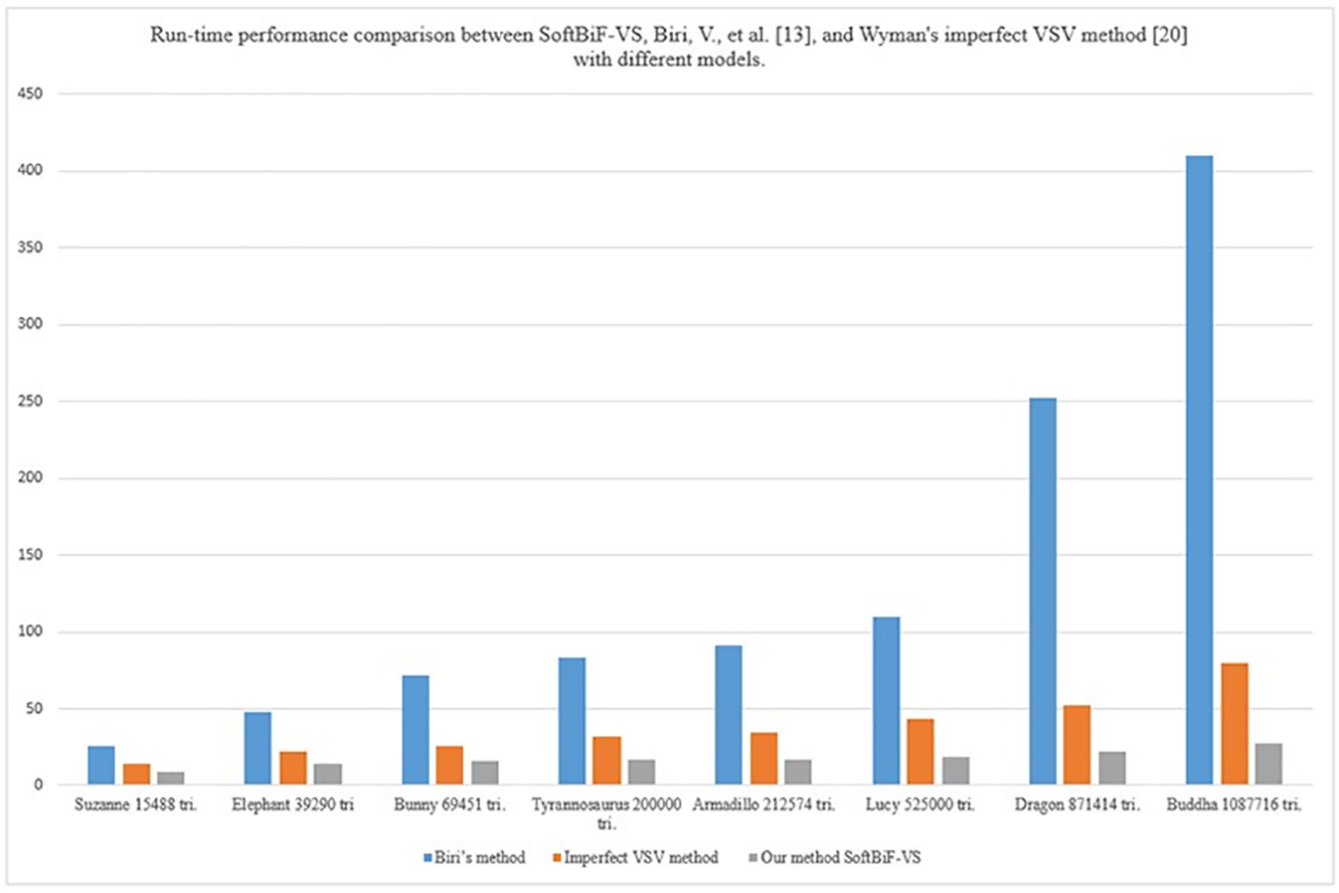
Frame rate of SoftBiF-VS technique compare with the Biri’s method [13], and Wyman’s Imperfect VSV method [20].

Fig 14 is plotted as a compares the run-time performance of SoftBiF-VS technique, Biri’s method [13], and Wyman’s Imperfect VSV method [20] for rendering the above models of 800×600 resolution. While SoftBiF-VS renders Suzanne (15488), Elephant (39290), Bunny (69451 tri.),Tyrannosaurus (200000), Armadillo (212574), Lucy (525000 tri.) Dragon (871414 tri.) and Buddha (10877716 tri.) models in 9.1 ms, 14.5 ms, 15.9 ms, 16.9 ms, 17.2 ms, 18.5 ms, 21.7 ms, and 27.03 ms respectively, the Biri’s method [13] renders Suzanne (15488), Elephant (39290), Bunny (69451 tri.),Tyrannosaurus (200000), Armadillo (212574), Lucy (525000 tri.), Dragon (871414 tri.) and Buddha (10877716 tri.) models in 25.6 ms, 47.6 ms, 71.4 ms, 83.3 ms, 90.9 ms, 109.4 ms, 252.6 ms, and 410.5 ms respectively, the Wyman’s Imperfect VSV method [20] renders Suzanne (15488), Elephant (39290), Bunny (69451 tri.),Tyrannosaurus (200000), Armadillo (212574), Lucy (525000 tri.) Dragon (871414 tri.) and Buddha (10877716 tri.) models in14.4 ms, 21.7 ms, 25.6 ms,32.3 ms, 34.5 ms, 43.5 ms, 52.6 ms, and 79.9 ms respectively. SoftBiF-VS shows the highly interactive rates, which is faster than Biri’s method [13] and Wyman’s Imperfect VSV method [20]. This indicates that our technique can render the complex scenes in real-time effectively.

## Conclusions and the future work

We proposed aSoftBiF-VS technique based on downsampling of ray marching to benefit from bilateral filtering. The technique exploits a cube shadow map and volumetric point light to ensure fast rendering. It could render volumetric shadows in all directions of the scene. As a result, it makes frame-rate rendering in high performance even when the scenes are complex. The contribution of SoftBiF-VS isrendering soft volumetric shadows using undersampling of a light texture with interpolated bilaterally for scenes with huge number of triangles in real time. Minimizing sample points of light texture reduced artifacts at the intersections between volumetric point light and a blocker by concept edge-preserving and noise-reducing filter for images. SoftBiF-VS improves the visual cues of soft volumetric shadows using non-linear filtering. The experiment results of the proposed technique are promising. Although the techniquecould reduce the rendering process to some extent, it still can be a feasible method because itcould improve the efficiency of graphics hard ware. In order to improve the visual quality of volumetric shadows in all directions, the technique of cube shadow map can be used. We have proposed an accurate solution to the general soft volumetric shadows sampling problem. In future works, we will combine the soft volumetric shadows with colored light shafts to improve the phenomenon volumetric shadows in light scattering for rendering more realistic scenes in real time.

## Supporting information

S1 File (DOCX)
