# Supplementary material for: Soft bilateral filtering volumetric shadows using cube shadow maps
Source: PLoS One. 2017 Jun 20;12(6):e0178415. doi: 10.1371/journal.pone.0178415 (PMC5478108; doi:10.1371/journal.pone.0178415)
Supplement: S1 File — (DOCX) [file pone.0178415.s001.docx]

$${}\left\{ \begin{aligned} \\ \end{aligned} \right.$$

$${}\sum{}$$

${}\left( \right){}^{{}}$**A. Processing of Fragment Shader**

To conduct the processing in fragment shader, firstly attenuation of the lighting is computed for this method relying on the ad-hoc manner to find the minimum of the sphere light. This is determined by looking up of simple sphere intersection. Second, the sampling is taken of the half resolution minimum depth to reconstruct the world position based on linear distance. Then, both starting and ending points are determined to compute the sphere light. Then ray marching technique is exploited at minimum number of the samples along the view rays by reducing the steps for each sample to compute volumetric shadows. The sampling of the virtual depth cube map is used to compute light point depth and the depth map value for the current position. The amount of light scattering is computed as discussed in Section 3.1, each fragment needed to be solved according to the Equation (4), current optical depth and shadows. Shader pseudocode is shown as following:

Where:

InvVP: is inverse view projection matrix

halfDepthTex: is texture of half depth

linearDepthParam: is linear depth parameters

viewPos: is view position

lightPos: is light position

ShadowCubeMapIndirectionTex: is texture of cube shadow map indirection

cubeShadowProj: is cube shadow projection

virtualShadowCubeMapTex: is texture of virtual cube shadow map

UV: is 2D texture

texCoord: is a texture coordinate

P: is value of pixel that computed using ray marching

pixeRayMarch: is a function to compute ray marching method

value2dTex: is value of 2D texture

d: is value of depth

depth2dTex: is depth of 2D texture

d0: is start of depth

d1: is end of depth

curPos: is current position of point in world space

viewPos: is view position

viewVecNorm: is normal of view vector

stepLen: is length stepping along view ray

numSteps: is number of steps along view ray

stepLenWorld: is length stepping in world space

l1: is light scattering for each pixel

lightPos: is light position

tScat: is value for each point along view ray

density: is density of participating media

curOpticalDepth: is current optical depth

tExt: is extinction coefficient for each point along view ray

lightPointDepth: is depth of light point

curPosDepth: is depth of current position

scatteredLightAmount: is amount of scattered light

Color: is color of light

| Pseudocode: Fragment Shader |
| --- |
| UV← texCoord  P← pixelRayMarch (value2dTex, UV)  d← Depth (depth2dTex, UV)  d0← startDepth  d1← endDepth  curPos ←viewPos + viewVecNorm*startDepth;  stepLen ←numSteps  stepLenWorld ←stepLen * (endDepth-startDepth)  for i $\in$ [0… numSteps] do  curPos += stepLenWorld * viewVecNorm  l1 ←light(lightPos, curPos) * stepLenWorld * tScat * density  curOpticalDepth *= exp(-tExt * stepLenWorld * density)  Cp←curPosDepth  shadow = (lightPointDepth>curPosDepth) ? 1.0 : 0.0  scatteredLightAmount += curOpticalDepth * l1 * shadow  i+=stepLen  end for  return (scatteredLightAmount*Color , curOpticalDepth); |
